# Analysis of patellofemoral arthrokinematic motion quality in open and closed kinetic chains using vibroarthrography

**DOI:** 10.1186/s12891-019-2429-z

**Published:** 2019-01-31

**Authors:** Dawid Bączkowicz, Krzysztof Kręcisz, Zbigniew Borysiuk

**Affiliations:** 1 Institute of Physiotherapy, Faculty of Physical Education and Physiotherapy, Opole University of Technology, Prószkowska 76, PL-45-578 Opole, Poland; 2 Institute of Physical Education, Faculty of Physical Education and Physiotherapy, Opole University of Technology, Prószkowska 76, 45-758 Opole, Poland

**Keywords:** Kinetic chain, Arthrokinematics, Vibroarthrography, Motion quality, Friction, Chondromalacia, Crepitus

## Abstract

**Background:**

Knee movements performed in open (OKC) and closed (CKC) kinetic chains generate various patterns of muscle activities and especially distinct contact stresses in the patellofemoral joint (PFJ). In contrast to these features, the arthrokinematic motion quality (AMQ) of the PFJ has not been compared between mentioned conditions. In this study we performed vibroarthrographic analysis of AMQ in movements performed in OKC and CKC, in healthy subjects and individuals with chondromalacia patellae, to assess which of the test conditions is more efficient in differentiation between healthy and deteriorated joints. Moreover, our analysis will broaden the knowledge related to behavior of normal and pathological synovial joints during motion with and without weight bearing. It is an essential issue, due to the recently observed significant interest in comparing potential benefits and limitations of CKC and OKC exercises as they relate to lower extremity rehabilitation.

**Methods:**

100 subjects (62 healthy controls and 38 subjects with PFJ chondromalacia) were enrolled. During repeated knee flexion/extension motions performed in OKC (in a sitting position) and CKC (sit-to-stand movements), the vibroarthrographic signals were collected using an accelerometer and described by variability (VMS), amplitude (R4), and spectral power in 50–250 Hz (P1) and 250–450 Hz (P2) bands.

**Results:**

Significant differences in VMS [V], R4 [V], P1 [V^2^/Hz] and P2 [V^2^/Hz] between OKC and CKC were found (0.0001, 0.969. 0.800 0.041 vs 0.013, 3.973, 6.790, 0.768, respectively, *P* < 0.001). Moreover, in both analyzed load-related conditions the subjects with chondromalacia were characterized by significantly higher values of all parameters, when compared to controls (*P* < 0.001), with effect size values over 0.6.

**Conclusions:**

We showed that motion of the physiological, unloaded PFJ articular surfaces in OKC is nearly vibrationless, which corresponds with optimal AMQ of PFJ, while loaded movements in CKC are characterized by a higher vibroacoustic emission level. Moreover, chondral lesions should be considered as an increased friction-related, aggravating factor of AMQ, which is critical in CKC movements under load. Nonetheless, OKC and CKC conditions are characterized by large effect sizes, and provide an efficient test frame for differentiating physiological knees and joints with chondral lesions.

## Background

The patellofemoral joint (PFJ), due to bone anatomy and the numerous capsuloligamentous structures and muscles that act dynamically on the patella, is considered one of the complex joints of the human body [1, 2]. A physiological adaptation of the PFJ to large weight bearing is the thick layer of hyaline cartilage (6–7 mm), which in physiological conditions provides nearly frictionless arthrokinematic motion [3–5]. Nonetheless, considerable involvement of the knee extension mechanism in daily activity causes generation of significant joint reaction forces, often leading to overloading of the PFJ structures [2–6]. These mechanisms related to excessive intra-articular stress and friction result in accelerating wear and degeneration of chondral structures, which may manifest as deterioration of both quantitative and qualitative aspects of osteokinematic and arthrokinematic motion [4, 7–9].

In contrast to quantitative assessment of joint motion via range of motion measurements using a goniometer or arthrometer, the qualitative analysis of joint motion has significant limitations. In clinical conditions this evaluation mainly focuses on palpation to assess integrity and smoothness of motion with regard to the presence or absence of crepitus. However, assessment of these mechanical vibrations and audible cracking or grinding sensations during articular motion is imprecise and highly subjective in nature (e.g. crepitus is often graded only as none, possible, or definite) [4, 10, 11]. Therefore, vibroarthrography (VAG) is suggested as a helpful tool supporting objective evaluation of arthrokinematic motion quality [12, 13]. Previously, the level of vibroacoustic emission has been shown to correspond closely with the degree of chondral deterioration [14]. It was reported that OA knees produce vibroacoustic emissions with a greater frequency, higher peaks, and longer duration compared to healthy knees [15–17]. However, recently it has also been demonstrated that the VAG method could be helpful in differentiating particular disorders of the PFJ and its stages, due to the specific, disorder-related character of the VAG signal pattern [4, 12]. It is believed that such analysis provides specific information about tribological properties of the joint, related among other factors to hyaline cartilage status and/or rheological characteristics of synovial fluid [18, 19]. Thus, the VAG method provides clinicians insight into mechanical, friction-reducing features of the articular environment, which reflects the overall condition of the joint, with special regard to its function [20]. This broadens the possibilities of physical examination and enables the selection of specific physical therapy interventions. It also seems that the VAG method may be a complement to the imaging methods (X-ray, MRI), where only structural changes are observed and articular function and qualitative aspects of joint motion are undetectable [4].

Although the VAG method is still in development, it shows high accuracy, sensitivity and specificity, when comparing results obtained from controls and joints with various disorders of different stages [4, 14, 15]. However, currently there are no strict guidelines for the use of specific research equipment (especially the kind of sensors), test conditions, data processing (the range of used frequencies, signal filtering) and parameters’ estimation, which may result in inconsistencies between outcomes. Some authors have applied analysis based on closed kinetic chain (CKC) movements, using repeated sit–stand–sit movements [16–18]. On the other hand, especially patients with patellofemoral syndromes often have difficulty with CKC regimens; therefore, many VAG analyses of PFJ-related disorders have been conducted in open kinetic chain (OKC) movements, without knee weight-bearing in a sitting position [19–21]. Nevertheless, from the biomechanical point of view, movements performed in CKC and OKC generate various patterns of muscle activities, ligament forces and particularly distinct PFJ contact stresses [22]. However, in contrast to these aspects, the direct impact of the applied loads related with the mentioned test conditions on the quality of PFJ arthrokinematic motion was not analyzed and remains unrecognized. It is also not known whether any of the CKC and OKC conditions provide a more efficient test frame to compare healthy joints and those with chondral lesions. Therefore, the purpose of the present study was to perform a vibroarthrographic comparative analysis of the PFJ arthrokinematic motion quality in unloaded OKC movements and CKC movements with weight bearing. The study included physiological knees and joints with chondral lesions, enabling us to assess which of the test conditions is more efficient in differentiation between healthy and deteriorated joints. We believe that these analyses will allow a new perspective on PFJ arthrokinematics and could broaden the knowledge related to joint tribology and especially the behavior of normal and abnormal synovial joints during loaded and unloaded motion. It is an essential issue, due to the recently observed significant interest in comparing potential benefits and limitations of CKC and OKC exercises as they relate to lower extremity rehabilitation [22–25].

## Methods

### Study population

One hundred volunteers (57 females and 43 males) aged 30 to 40 years were enrolled in the study. Participants were classified into two groups: 62 healthy control subjects (CTRS) and 38 individuals with chondromalacia patellae (ChMP). Age, gender and anthropometric data of subjects in the analyzed groups are given in Table 1.

**Table 1:** Characteristics of analyzed groups

	*n*	Males/Females	Age [years]	Height [cm]	Weight [kg]
			mean (SD)	mean (SD)	mean (SD)
All participants	100	43/57	35.3 (6.3)	168.4 (7.8)	69.8 (12.3)
Healthy controls (CTRS)	62	27/35	35.5 (5.9)	168.4 (7.4)	69.2 (13.1)
Patients with chondromalacia (ChMP)	38	16/22	34.9 (6.5)	168.1 (7.2)	69.2 (13.1)

*Abbreviations*: *n* number of cases

The clinical evaluation of the CTRS group was based on both anamnesis (participant’s self-reported medical history) and physical examination performed by a senior physiotherapist, but without radiological exclusion of the cartilage pathologies. Only individuals with no history of knee disorders or other diagnosed injury or pathology within the lower extremity were included in the control group. Symptomatic patients with stage II and III of isolated ChMP were enrolled in the ChMP group, which means that the individuals were characterized by the presence of blister-like swelling/fraying of PFJ articular cartilage extending to the surface with less than 50% (stage II) and more than 50% thickness cartilage loss with focal ulceration (stage III). The cases were diagnosed by MRI imaging in accordance with the modified Outerbridge classification [26, 27], determined by an independent radiologist blinded to patients’ symptoms. To prevent any signal artifacts originating from the influence of factors not associated with ChMP, cases with other types of chondral lesions, ligament/tendon ruptures, functional limitations (restriction in range of knee joint motion, significantly weakened quadriceps muscle, problems with performing a squat) and acute inflammation were excluded from the study. Moreover, because previously it has been shown that patellar malalignment leads to increased compression as well as friction between the patella and condyles of the femur, if in the MRI image translational or rotational deviation of the patella was noted, the analyzed knee joint was also excluded from the study, to prevent patella maltracking influencing the results [4].

For all participants only one knee joint was analyzed – in the CTRS group a randomly selected knee and in the ChMP group only a knee which satisfied the inclusion/exclusion criteria. If both lower limbs of the ChMP patient had similar involvement, the knee less symptomatic or preferred by the patient was assessed.

### Assessment of arthrokinematic motion quality

Based on the previous studies, assessment of the PFJ arthrokinematic motion quality was performed with an accelerometer sensor placed, in a seated position, 1 cm above the apex of the patella [4, 12, 28]. Measurement was performed in repeatable motion sequences (90°-0°-90° of knee flexion) in the CKC and OKC conditions.

**Fig. 1:**
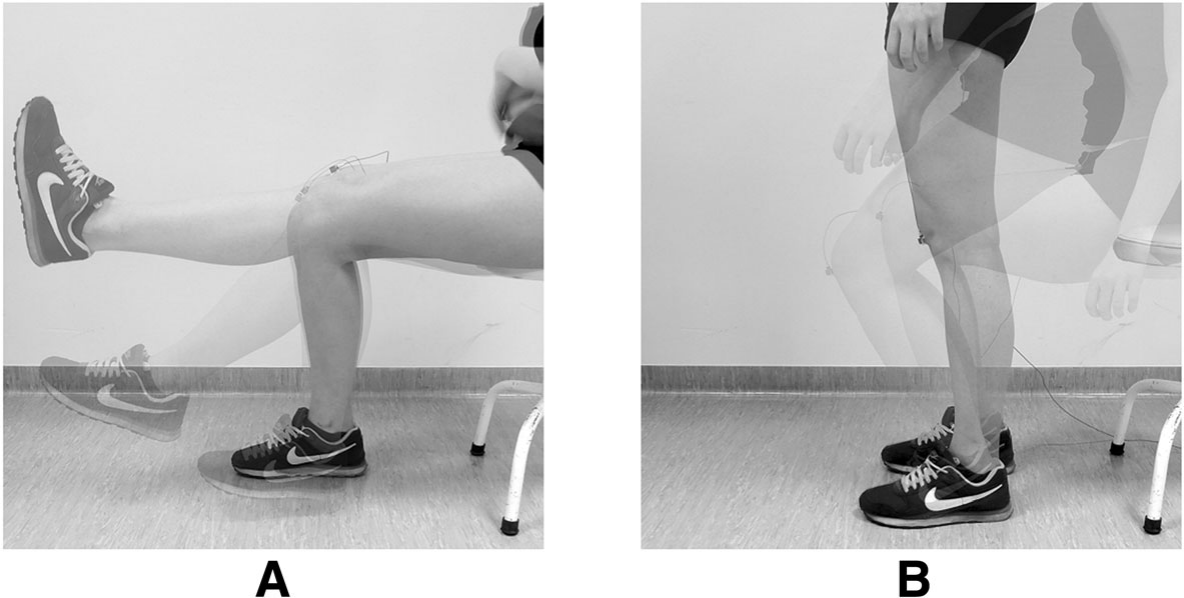
Assessed motion sequences in OKC (**a**) and CKC (**b**) conditions. Source: Author’s own figure

For the CKC analysis, the following procedure was performed: (i) ascending from a sitting position on the chair with knee flexed to 90°, (ii) reaching a fully erect standing position (0° of knee flexion); (iii) descending to return to a seated position (90° of knee flexion). In the OKC assessment was based on: (i) loose hanging legs with knees flexed at 90°; (ii) full knee extension from 90° to 0°; (iii) re-flexion (from 0° to 90°) in a sitting position (Fig. 1).

In both conditions, the constant velocities of flexion/extension movements were kept at 82 beats per minute with a metronome. In total, participants were asked to perform 4 sit–stand–sit (CKC) and 4 flexion-extension-flexion sequences (OKC), each series in a 6-s period, with one 1-min break between series. The order of the CKC and OKC series was randomly selected.

In the preliminary examination (to demonstrate and verify the correctness of the movements of each participant), the range of knee flexion-extension motion was tracked using an electrogoniometer. However, because of the high sensitivity of the accelerometer used, the VAG signal was distorted by the noise generated by electrogoniometer, and so in relevant tests it was taken off. The height of the seat was adjusted to the height of the participants, such that the lateral aspect of the knee was lined up with the axis of rotation at femoral condyle.

The VAG signals were collected using an acceleration sensor, Brüel & Kjær model 4513B-002, with a multi-channel Nexus conditioning amplifier (Brüel & Kjær Sound & Vibration Measurement A/S, Denmark). Data were recorded at sampling frequency of 10 kHz and then filtered using a fourth-order zero-phase Butterworth band-pass digital filter with cutoff frequencies between 50 Hz and 1000 Hz. The variability of the VAG signal in the time domain was assessed by computing the following parameters [4, 12]:

1. the mean-squared values of an obtained signal in fixed-duration segments of 5 ms each and then computing the variance of the values of the parameter over the entire duration of the signal (VMS) [29];
2. signal amplitude was calculated as the difference between the mean of the four most prominent peaks and the mean of the four most prominent troughs of the VAG signal (R4).

The frequency characteristics of the VAG signal were examined by a short-time Fourier transform analysis. The short-time spectra were obtained by computing the discrete Fourier transform of segments, 150 samples each, Hanning window, and 100 samples overlap of each segment. The spectral activity was analyzed by summing spectral power of the VAG signal in two bands: 50–250 Hz (P1) and 250–450 Hz (P2) [4, 12, 13, 28].

### Statistical analysis

Normality of the distribution of the VAG signal parameters was assessed using the Shapiro-Wilk test and visual inspection. Additionally, Levene’s test for equality of variances was conducted. Due to skewed distributions and lack of heterogeneity of variances of each parameter, Box-Cox transformation was applied [30]. Evaluation of all dependent variables was subjected to 2 groups (CTRS and ChMP) × 2 conditions (OKC and CKC) analysis of variance (ANOVA) with repeated measures of the last factor. When significant interactions were identified, Tukey’s HSD for unequal sample sizes follow-up analyses were applied as post-hoc tests. Effect size was calculated as 0.642(Y2-Y1)/Sw, where Yj is the population 20% trimmed mean for the jth level of the grouping factor and Sw is the square root of the pooled 20% Winsorized variance [31].

ANOVA and post-hoc tests were performed using Statistica v.13.1 (StatSoft, Inc., OK, USA), while the effect size was calculated using the walrus R package (https://github.com/jamovi/walrus) with the median M-estimator method for group comparison.

## Results

**Fig. 2:**
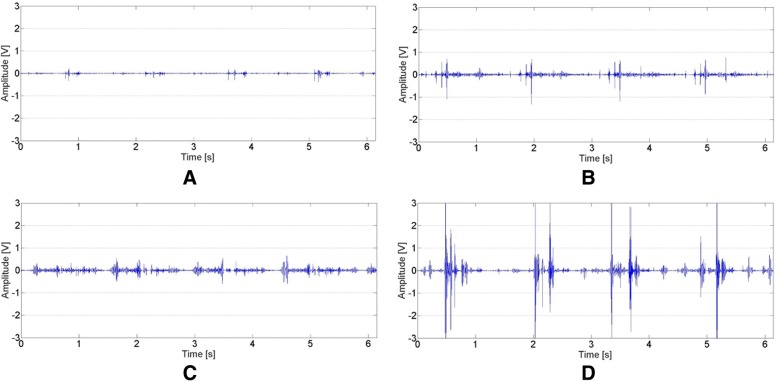
Plots of VAG signals representative for CTRS group recorded in OKC and CKC (**a**, **b**) and for ChMP group recorded in OKC and CKC (**c**, **d**)

**Fig. 3:**
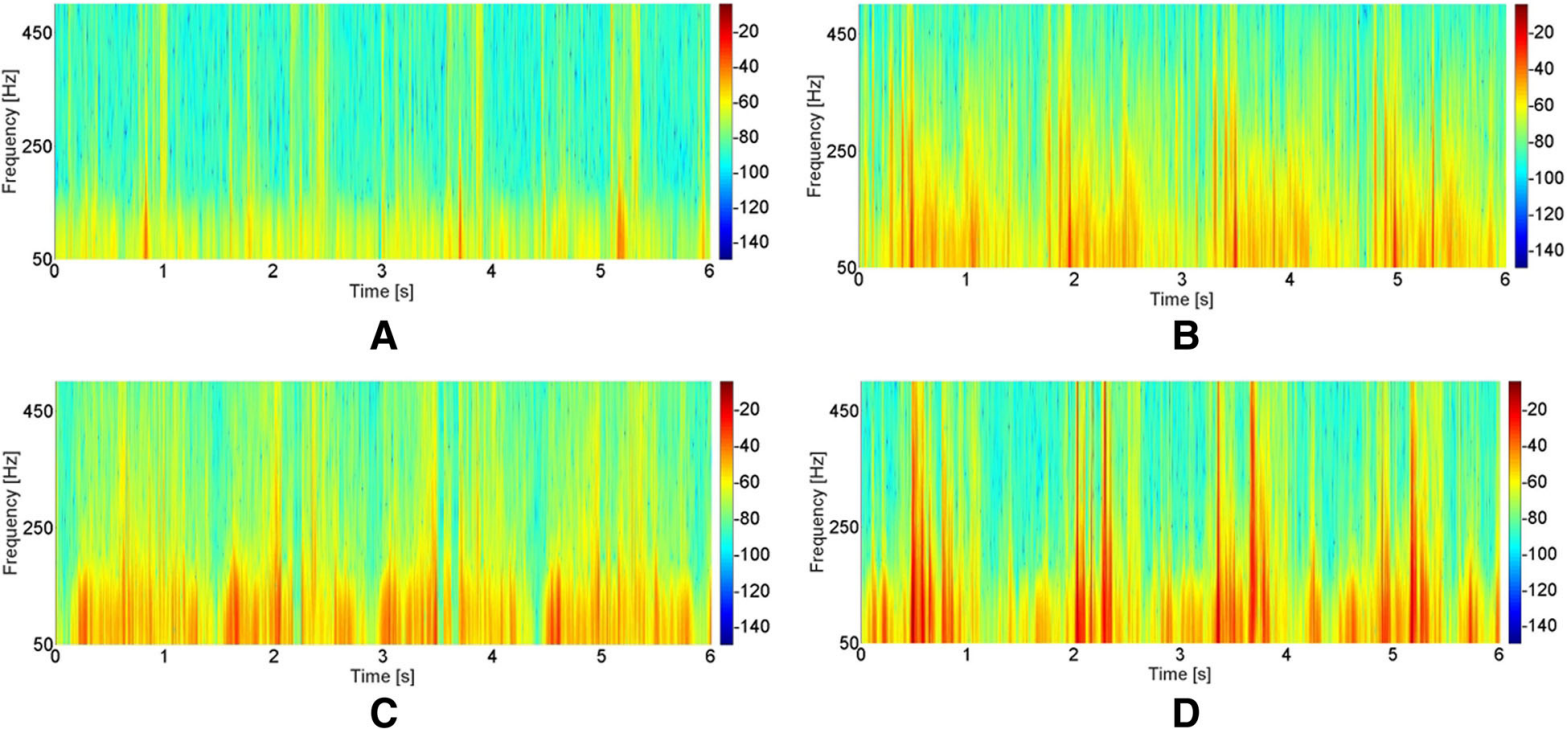
Time-frequency representations of VAG signals representative for CTRS group recorded in OKC and CKC (**a**, **b**) and for ChMP group recorded in OKC and CKC (**c**, **d**)

The obtained data are presented in Table 2 and Fig. 2 as back-transformed medians and quartiles from Box-Cox transformations (λ = 0.00605 for OKC VMS, λ = 0.063104 for OKC R4, λ = 0.074237 for OKC P1, λ = − 0.062911 for OKC P2, λ = 0.014114 for CKC VMS, λ = 0.181420 for CKC R4, λ = − 0.059244 for CKC P1, λ = 0.081630 for CKC P2). Outcomes of performed statistical analysis (main effects and interactions of ANOVA) are presented in Table 3. Moreover, for better interpretation of presented data, we have provided sample plots of recorded VAG signals (Fig. 2) and their respective spectrograms presenting the distribution of the signal frequency spectrum (Fig. 3), representative for each group analyzed in OKC and CKC conditions.

**Table 2:** Values of back-transformed values of VAG parameters for analysis performed in open (OKC) and closed (CKC) kinetic chains

		CTRS, *N = 62*			ChMP, *N = 38*			effect size
		lower quartile	median	upper quartile	lower quartile	median	upper quartile	
VMS [V]	OKC	0.00000	0.00002	0.00004	0.00031	0.00082	0.00288	0.714
	CKC	0.00046	0.00278	0.01493	0.02081	0.26602	0.64750	0.677
R4 [V]	OKC	0.46200	0.62300	0.83700	1.47200	1.77900	2.45900	0.872
	CKC	1.79000	2.44000	3.94000	5.22000	7.47000	9.44000	0.785
P1 [V^2^/Hz]	OKC	0.24900	0.39700	0.65700	1.37900	2.35700	3.86300	0.883
	CKC	1.82000	3.18000	7.47000	6.29000	22.4500	36.99000	0.654
P2 [V^2^/Hz]	OKC	0.01090	0.01800	0.03830	0.07590	0.14100	0.23450	0.926
	CKC	0.13000	0.29500	0.91400	1.35600	2.43400	4.79500	0.741

*Abbreviations*: *CTRS* healthy subjects, *ChMP* patients with chondromalacia, *VMS* variance of the mean squares calculated in 5 ms windows; R4, the difference between the mean of four most prominent peaks and the mean of four most prominent troughs of VAG signal; P1, P2, power spectral density bands: 50–250 Hz and 250–450 Hz, respectively; N, number of analyzed knees

**Table 3:** Main effects and interactions of ANOVA for analyzed VAG parameters after Box-Cox transformation

	Effect of kinetic chain		Effect of group		Kinetic chain x group interaction	
	F(1.98)	*P*	F(1.98)	*P*	F(1.98)	*P*
VMS [V]	328.94	< 0.001	148.36	< 0.001	2.08	0.152
R4 [V]	392.56	< 0.001	144.29	< 0.001	0.10	0.756
P1 [V^2^/Hz]	264.76	< 0.001	118.39	< 0.001	4.71	0.032
P2 [V^2^/Hz]	429.41	< 0.001	102.48	< 0.001	2.60	0.110

VMS, variance of the mean squares calculated in 5 ms windows; R4, the difference between the mean of four most prominent peaks and the mean of four most prominent troughs of VAG signal; P1, P2, power spectral density bands: 50–250 Hz and 250–450 Hz, respectively

The obtained results show that in the OKC condition, when evaluating the CTRS group, recorded signals possess a “smooth” course with slight cyclical growth of intensity only (Fig. 2a). These features are confirmed by low values of VMS and R4 parameters (Table 2). Moreover, it can be observed that the unloaded motion of physiological PFJ generates a signal concentrated mainly up to 250 Hz (Fig. 3a), which is described by the P1 parameter. In comparison to controls, knees with chondromalacic changes, also evaluated in OKC, generated signals with significantly higher variability, amplitude and higher spectral power at 50–250 Hz and 250–450 Hz (Figs. 2c and 3c). These differences are clearly noticeable for variance of the VAG signal course (VMS), where the median in the ChMP group is over one order of magnitude higher than in controls (Table 2).

VAG signals acquired during CKC movements were characterized by a much higher level of vibration emission than in OKC, both in CTRS and ChMP groups (Fig. 2b and d). It is reflected by statistical comparison between OKC and CKC conditions, which reveal significant differences in all investigated parameters (Tables 2 and 3). The mentioned phenomenon is clearly visible in Fig. 2b, where plots of recorded signals typical for healthy control knees analyzed in CKC are characterized by replicable peaks, in each of four cycles of motion.

**Fig. 4:**
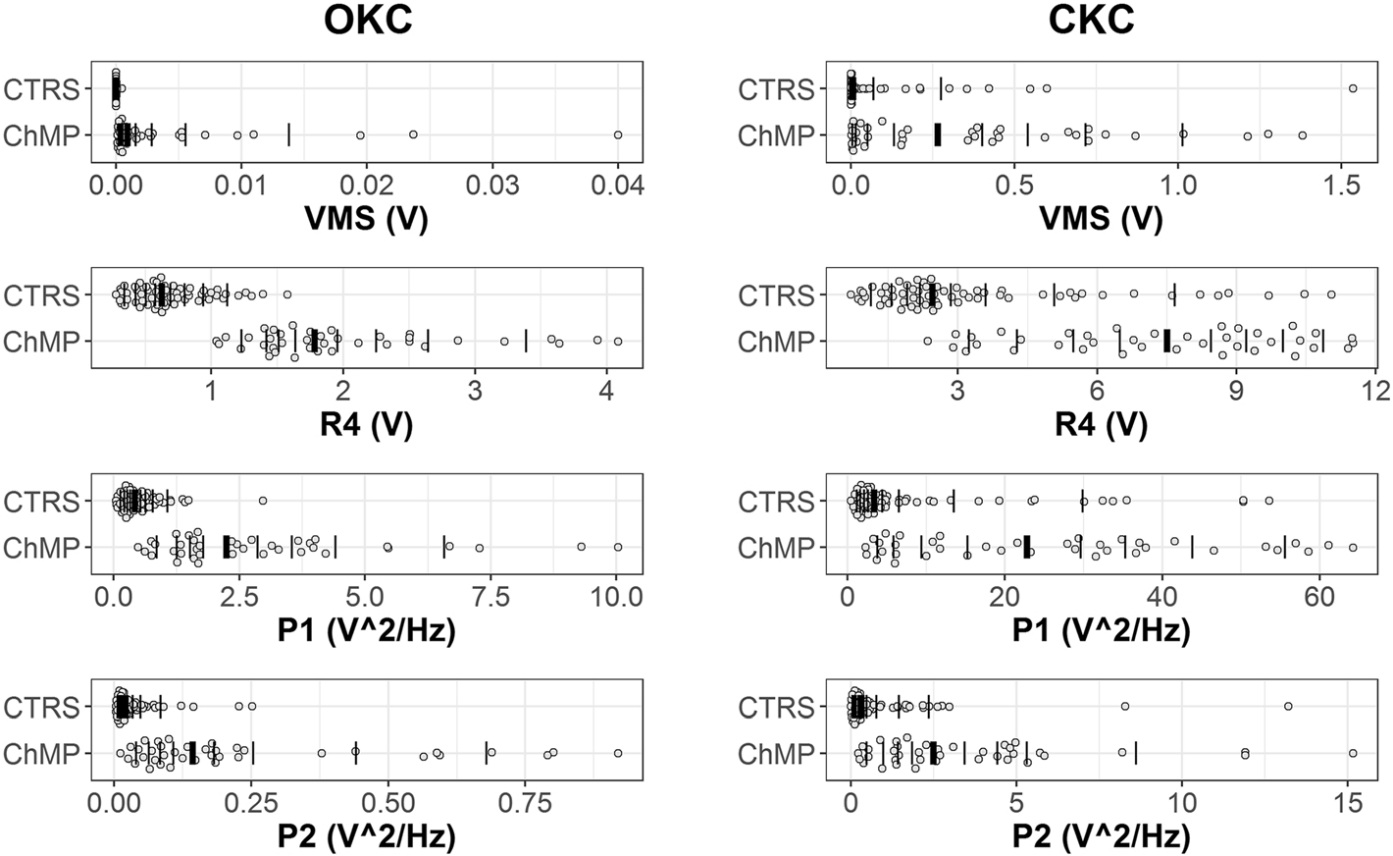
Dot plots of all extracted features in the OKC and CKC conditions with vertical lines marking the deciles for each group. The thicker vertical line in each distribution is the median. (Plots created using the rogme R package: https://github.com/GRousselet/rogme)

A similar VAG signal pattern, but on a much larger scale, is observed for the ChMP group. Performed movements generate prominent vibrations, noticeable in the VAG signal course as multiple high-amplitude peaks (Fig. 2d) with high energy (Fig. 3d). It results in the highest values of all analyzed parameters described in registered VAG signals, which are several times higher than in the CTRS group in the same condition (Fig. 4). It should also be noted that comparison of groups reveals effect sizes with values over 0.6, in both conditions (Table 2). Nonetheless, for each VAG parameter, the values of the effect size in OKC are slightly higher than that in the CKC condition, and the largest difference was found for the P1 and P2 parameters.

## Discussion

The aim of the present study was to assessment of PFJ arthrokinematic motion quality in two biomechanically different conditions: OKC movements performed without weight load and CKC movements with weight bearing. Moreover, in order to compare the appropriateness of the two mentioned test conditions for the recognition of chondral lesion-related arthrokinematic deteriorations, our analyses were based on the acquisition of the vibroacoustic emission from the physiological PFJ and joints with stage II and III of ChMP. We assumed that the intensity of vibroacoustic emission waves is closely connected with the kinetic friction of synovial joints and corresponds to the arthrokinematic motion quality [4, 14, 32].

The results presented herein showed that the movements performed in OKC and CKC conditions otherwise affect the PFJ arthrokinematic motion quality analyzed by the VAG method. We observed that motion of the unloaded physiological articular surfaces is nearly vibrationless, which from the biomechanical and tribologic points of view indicates optimal arthrokinematic motion quality [12, 13]. This observation is in agreement with the thesis that physiological synovial joints, mainly through biomechanical properties of the superficial tangential zone of hyaline cartilage and synovial fluid, possess an extremely low coefficient of friction, ~ 0.002–0.004, which is the ratio of the friction force during sliding divided by the applied load [3, 33]. For comparison, lubricated PTFE (Teflon) sliding on PTFE has a coefficient of friction of about 0.04, an order of magnitude higher than that for synovial joints [5]. The phenomenon observed by us may also result from the fact that knee flexion-extension movement in OKC without a load generates relatively low contact stress between PFJ surfaces. In an experimental study on cadaveric knees, it was found that during unloaded motion from 20° to 90° of knee flexion, the values of PFJ contact stress ranged only from 0.4 to 0.7 MPa [34, 35].

Previous vibroarthrography-related research also performed in OKC showed that articular surfaces with chondral lesions possess diminished capabilities of friction reduction and generate a distinct VAG waveform pattern compared to those from healthy joints [12, 20, 21]. Accordingly, in our study, knees with chondromalacic changes were characterized by signals with higher amplitude and variation, when compared to the CTRS group. It was also confirmed in power spectral analysis, showing escalated participation of high frequency (50–450 Hz) in signals recorded from knees of patients with chondromalacia. This occurence seems to be related to biomechanical and morphological changes within softened hyaline cartilage – in stage II and III of chondromalacia the deeper chondral layers were already exposed. These layers, when compared with the superficial zone, possess distinct structure and function. Collagen fibers are loosely packed in oblique and vertical orientations to ensure self-amortization, but not for reduction of kinetic friction [36]. Therefore in chondral lesions with exposure of inner layers, the cartilage surface becomes rough, which results in deteriorated arthrokinematic motion quality [4, 37].

In the movements with weight load (CKC), the PFJ arthrokinematic motion quality was characterized by significantly higher values of all VAG parameters, in comparison to the OKC condition. This phenomenon, observed in both analyzed groups (CTRS and ChMP), may be justified by the fact that in weight loaded CKC movements the PFJ contact stress is much larger than in OKC. It was previously observed in cadaveric and computer modeled studies that during squatting, due to the quadriceps forces and extensor lever arm, the PFJ contact stress is about 16-fold higher than in OKC, and reaches 11.1 MPa at 90° of knee flexion [22, 34]. Thus it seems that high contact stress determines the increase of kinetic friction, which is observed in the VAG method as the signal course possessing higher variability and amplitude as well as being characterized by higher power spectral density than that in OKC. The mentioned occurrence is especially visible in knees with chondromalacic changes, indicating that articular surfaces with deteriorated chondral layers possess limited possibilities of friction reduction and in movements performed under load generate prominent vibrations. This observation may explain why many patients with PFJ chondral deterioration experience audible and palpable popping, snapping or grinding sensations that arise often during getting up from a sitting position. But it should be noted that the load-related intensification of vibroacoustic emission is a physiological process and also occurs within healthy joints, although on a much smaller scale.

The clinical significance of our findings can be discussed alongside the results of those studies that have shown that the pattern of vibroacoustic emission corresponds to the degree of articular environment deteriorations [4, 12, 13]. Our findings confirmed that in contrast to the methods of the direct analysis of PFJ contact forces or arthrokinematic friction (due to the typical laboratory character of these methods using cadaveric specimens or computer models), the VAG assessment may be a clinically useful tool for assessment of PFJ arthrokinematics [37]. Nevertheless, to the best of our knowledge, our paper is the first publication which shows that the quality of arthrokinematic motion analyzed by the VAG method depends on the level of the PFJ load and seems to be related to PFJ contact stress. Moreover, despite the distinctions discussed above, our results showed that both analyzed load-related conditions are characterized by large effect sizes, and provide an efficient test frame for differentiating physiological knees and joints with chondral lesions. Nonetheless, it should be noted that for each VAG parameter, the values of the effect size in OKC were slightly higher than that in the CKC condition. Observed differences seem to be clinically meaningful, because when combined with the frequently occurring functional restrictions of patients with PFJ deteriorations (e.g. problems with performing a squat) and greater ease of performing unloaded movement, it may indicate that the OKC is more suitable for quantifying the differences between healthy joints and those with chondral lesions within the anterior knee compartment. This finding may also be applied to clinical practice as a deeper analysis of palpation/crepitus occurring in patients with chondral disorders, e.g. ChMP or osteoarthritis [4, 12].

However, it should be noted that our analyses were based only on the two test conditions (OKC vs CKC), which is a limitation in drawing a conclusion about the impact of the load on the PFJ arthrokinematic motion quality. Thus, we propose that in subsequent studies the impact of progressive load should be analyzed, similarly to the evaluation of patellofemoral stresses presented by Cohen et al. [34]. Moreover, it should be noted that the VAG method is fraught with some limitations, the most pronounced being the indirect acquisition of the vibroacoustic signal, using skin-mounted accelerometers. Thus, the raw time series may present not only vibrations related to motion of articular surfaces, but may contain noise from muscle and skin vibrations. Because of this multicomponent character of the VAG signal, high-pass filtering was performed at the 50-Hz threshold to limit the impact of artifacts. Therefore, it should also be taken into account that certain anthropometric characteristics related to interpersonal anatomical differences (skin elasticity, fat tissue thickness) could have a slight impact on the obtained results. Nonetheless, we showed that the chondromalacic changes within PFJ result in dramatic deterioration of arthrokinematic motion quality (observed as a higher level of vibroacoustic emission), especially during knee movements under a weight load. This phenomenon should be considered when planning physical activities and rehabilitation strategies for patients with PFJ chondral lesions, because excessive friction may lead to accelerated abrasion of the articular cartilage [38, 39].

## Conclusions

The presented VAG analysis indicated that the quality of arthrokinematic motion not only depends on the condition of the articular cartilage, but also is related to the performed task and the related level of the joint load. We showed that knee flexion-extension movements performed in unloaded OKC movements and CKC movements with weight bearing are associated with distinct levels of vibroacoustic emission, which seems to be related to PFJ contact stress and kinetic friction. Nonetheless, although OKC and CKC are biomechanically different conditions, both of them are efficient for quantifying the differences between healthy joints and those with chondral lesions. However, due to the frequently occurring functional limitations of patients with PFJ deteriorations and greater ease of performing unloaded movement, the OKC condition seems to be a more suitable test frame for vibroarthrographic analysis of the anterior knee compartment.

## Abbreviations

ChMP: Chondromalacia patellae, group of patients with chondromalacia
CKC: Closed kinetic chain
CTRS: Control group
OKC: Open kinetic chain
PFJ: Patellofemoral joint
VAG: Vibroarthrography
